# Navigating the Strengths and Constraints of Mouse Models in Obesity Research

**DOI:** 10.1210/endocr/bqaf123

**Published:** 2025-07-24

**Authors:** Patric J D Delhanty, Jenny A Visser

**Affiliations:** Department of Internal Medicine, Erasmus MC, University Medical Center Rotterdam, Rotterdam 3015 GD, The Netherlands; Department of Internal Medicine, Erasmus MC, University Medical Center Rotterdam, Rotterdam 3015 GD, The Netherlands

**Keywords:** mouse models, monogenic obesity, sex differences, thermogenesis, cross-species conservation

## Abstract

Obesity is a major health problem, being a risk factor for many metabolic diseases. Obesity results from an imbalance in energy intake and energy expenditure. Animal models, particularly naturally occurring mouse models of obesity, have provided a framework of the basic mechanisms regulating energy homeostasis. However, there remain gaps in our understanding of the mechanisms underlying the pathophysiology of obesity. Mouse models of obesity remain an essential tool to further our knowledge, due to advanced tools for genetic manipulation and the possibility to study interaction with environmental factors, such as diet. While there are advantages to using mice as models of obesity, it should be recognized that there are limitations. In this mini-review we provide a brief overview of the monogenic mouse models of obesity that have led to the discovery of important physiological systems that regulate energy homeostasis, such as the leptin-melanocortin pathway, that translate well to humans. We also discuss confounding factors that, when taken into account, might improve translatability of these findings. Finally, we discuss potential strategies to determine functional consequences of non-coding genome-wide association study (GWAS) signals in mouse models.

According to the World Health Organization, globally, 1 in 8 people are obese (1). Obesity is caused by an imbalance in energy intake and energy expenditure leading to an increase in fat mass. It is associated with many chronic health conditions, including type 2 diabetes, cardiovascular disease, several cancer types, and infertility. As such, obesity is a major health concern. A sedentary lifestyle and obesogenic environment partly explain the rise in obesity prevalence (2, 3). However, obesity is considered a multifactorial disease and the interplay between genetic and environmental factors will determine an individual's susceptibility to develop obesity (4). Understanding the mechanisms involved in regulation of energy homeostasis is important to develop targeted treatments to combat obesity.

Over the years, mouse models have been instrumental in unraveling the pathways underlying control of energy homeostasis and have shown that the mechanisms regulating satiety and satiation are highly conserved across species (5). The leptin-melanocortin signaling pathway, in particular, has been identified as being important, as naturally occurring mutations in the leptin and leptin receptor gene explained the severe obesity of the *ob/ob* and *db/db* mice, respectively (6-9). Subsequently, mutations in components of this pathway were shown to cause severe obesity in humans as well (reviewed in (10, 11)). Furthermore, these experiments of nature have highlighted a neuroendocrine regulation of appetite and energy homeostasis, with a key role for the hypothalamus. These spontaneous mouse models of obesity, as well as genetically engineered mouse models, have significantly advanced our understanding of the biology of obesity. However, they only represent a small proportion of human obesity. While the heritability of body mass index (BMI) is high, being estimated at 40% to 70% based on twin and family studies (12, 13), monogenic obesity only explains a small percentage of obesity in humans. Depending on the study population, 0.8% to 30% of cases have been reported to be monogenic (14), underscoring that predisposition to common obesity has a polygenic basis. Polygenic mouse models may therefore more accurately reflect the multifactorial nature of obesity in humans. Such models may also be useful in unraveling the effect of environmental factors, including epigenetics, dietary challenges, and industrial pollutants. The advantages of mouse models in obesity research are the ability to control genetic background and environmental conditions. Combined with the possibility of advanced genetic manipulation, these models can provide unique insights into the genetic and environmental factors regulating energy homeostasis and obesity. While there are advantages to using mice as a model of obesity, it should be recognized that there are limitations in translatability of findings to humans.

This mini-review is not intended to be exhaustive but rather to highlight examples of how mouse models have contributed to understanding mechanisms of obesity and to provide directions for future research. First, we describe the most commonly used genetic and diet-induced models of obesity, summarized in Fig. 1. Next, we discuss examples of confounders that could affect the translatability of using mice, for example sex-dimorphic effects and differences in rates of energy expenditure. Finally, because a majority of current data relating to genetic mechanisms behind human obesity point to non-coding signals in the genome, we discuss recent technological advances to identify interspecies conservation of such signals. These could be leveraged to select variants with the highest potential for translatability to humans for study in mice.

**Figure 1.**
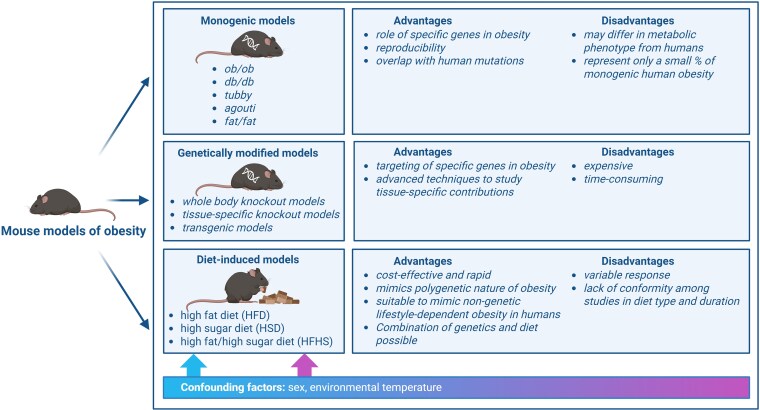
Schematic overview of mouse models of obesity. The main advantages and disadvantages of monogenic, genetically modified, and diet-induced mouse models of obesity are shown. In addition, confounding factors discussed in this review impacting obesity development in mice are shown. Created in BioRender. Visser, J. (2025) https://BioRender.com/ezsrmpu.

## Monogenic Mouse Models of Obesity

The leptin-melanocortin system pathway constitutes important neural circuits involved in metabolic homeostatic regulation. Hypothalamic neurons express the leptin receptor, and upon leptin stimulation, pro-opiomelanocortin (POMC)-expressing neurons produce α-melanocyte stimulating hormone (α-MSH). These hormones activate melanocortin-4 receptor (MC4R), resulting in decreased food intake. Under fasting conditions leptin levels decrease and neurons are activated to produce agouti-related peptide (AgRP), which antagonizes MC4R signaling, thereby stimulating food intake (15). In addition, the leptin-melanocortin system has been implicated in the regulation of hedonic (reward) aspects of food intake (16). Naturally occurring mouse models of obesity have been instrumental in identifying the leptin-melanocortin system as a major regulatory pathway in energy homeostasis. These mice carry a spontaneous monogenic mutation causing obesity and through positional cloning the genes underlying the obese phenotype were identified.

One of the first models found to spontaneously develop obesity is the obese mice line (*ob/ob*), discovered in 1949 at the Jackson Laboratory (17, 18). These mice display early-onset morbid obesity with mild insulin resistance, associated with hyperphagia and hyperglycemia (17). These mice become obese even when pair-fed (19, 20). With pair-feeding, the experimental group receives the same amount of food as consumed by the control group, which allows determination of whether effects on body weight occur independent of changes in food intake (21). Although this is a more elegant way to assess energy expenditure, most studies use ad libitum access to food. The pair-fed results obtained in the *ob/ob* mice suggested that their metabolic efficiency is increased, which is explained by their reduced thermogenesis as a result of a failure to activate their brown adipose tissue (BAT) (20, 22). The diabetes (*db/db*) mice closely resemble the *ob/ob* mice, except that they are more prone to develop diabetes (6). Parabiosis (cross-circulation) experiments between *ob/ob* and wild-type mice suggested that a circulating factor could rescue the *ob/ob* phenotype. In contrast, parabiosis between *db/db* and wild-type mice, did not rescue the obese phenotype, but rather induced hypophagia in the wild-type mice (23, 24). In the 1990s, it became evident that the affected genes in both mouse models are part of the same signaling pathway. The *ob* gene encodes for leptin, while the *db* gene encodes for its receptor that signals through the JAK-STAT pathway (7, 9, 25). Leptin is a factor secreted primarily by adipocytes and regulates energy balance by acting on the brain. Both peripheral administration and intravenous injections of leptin regulate appetite and metabolism through activation of hypothalamic neurons (26). Although initially considered an anti-obesity signal, the current view is that leptin acts as a signal of energy deficit (27). This is because decreasing levels of leptin are a key indicator of starvation in mice as well as humans. However, the obverse, that overfeeding-induced increased leptin levels prevent obesity development, has not been experimentally determined (27). This is also complicated by the fact that diet-induced obesity in mice results in leptin resistance, recently suggested to be caused by chronic mTOR activation in POMC neurons that reduces leptin signaling (28).

The *tubby* mice, which carry a mutation in the gene *Tub*, develop late-onset obesity and vision and auditory deficits (29-32). Generated *Tub*-deficient mice are a phenocopy of the *tubby* mice suggesting that these mice carry a loss-of-function mutation (33). *Tub* is expressed in several hypothalamic regions involved in energy homeostasis and in adipose tissues (34). Tubby is a member of the tubby-like family of proteins and may act as a bipartite transcription factor (35). Although the function of Tub remains to be fully deciphered, studies suggest that Tub also plays a role in G protein–coupled receptor (GPCR) trafficking to neuronal cilia. Studies in *C. elegans* and mammalian cells, suggest Tub-regulated ciliary trafficking of GPCRs is restricted to a subset of GPCRs (36, 37). It was shown that Tub regulated trafficking of Sstr3, Mchr1, and Npy2r, but not Gpr161 and Gpr19 (36, 37). In addition, translocation of Tub from the plasma membrane to the nucleus in response to activated GPCR signaling has been proposed, specifically upon activation of GPCRs that couple to Gα_q_ (38). It is not known whether this is a response to any Gα_q_-linked GPCR or to a specific GPCR. Its involvement in GPCR trafficking and/or signaling in primary cilia is intriguing since many ciliopathy cases are accompanied by obesity (39). Despite its implication in several signaling pathways, the phenotype of the *tubby* mice is milder than observed in *ob/ob* and *db/db* mice (29, 33). In humans, a mutation in *TUB* has been identified in siblings from a consanguineous family with early-onset obesity and retinal dystrophy (40). However, only mild obesity was observed in a patient carrying a different *TUB* mutation (41). Thus, the role of TUB in human energy homeostasis has not been fully elucidated and requires further study.

The *agouti* mice, or lethal yellow mutant mice (Ay), have implicated the melanocortin pathway in the regulation of energy homeostasis. These mice have a yellow fur and develop adult-onset obesity and moderate hyperphagia (42). *Agouti* was identified as the underlying gene, which is expressed in follicular melanocytes, and its product agouti signaling protein (ASP) antagonizes melanocortin-1 receptor (MC1R) in melanocytes by competing for α-MSH (43-46). However, the mutation, a deletion of 120-170 kb genomic DNA, results in ubiquitous expression of *agouti* (47). Subsequently, ASP, having affinity for multiple melanocortin receptors, now also can antagonize α-MSH binding to MC4R in the hypothalamus (45). As such, ASP mimics the function of its hypothalamic homologue AgRP (48). Indeed, transgenic overexpression of AgRP in mice also leads to development of obesity and hyperinsulinemia (49). The involvement of MC4R in regulating energy metabolism was confirmed with the generation of Mc4r knockout mice. These mice have a normal coat color but develop late-onset obesity with hyperphagia (50). *MC4R* mutations are the most common cause of monogenic obesity in humans, but, in contrast to mice, human *MC4R* mutations lead to an early onset of obesity (11, 51). The cause of this difference in timing of onset, also observed for some of the other obesity mouse models, is unclear. However, a more detailed study of Mc4r-deficient mice incorporating younger ages, suggests that excessive fat deposition may occur already at weaning without initially affecting body weight (52), suggesting obesity may develop or present differently during the life course in mice compared to humans.

Mutations that affect the processing of α-MSH have also been identified to result in obesity. α -MSH is derived from POMC, which is processed through a series of enzymatic steps by prohormone convertase 1 (PC1/3) and carboxypeptidase E (CPE) enzymes into ACTH, which is subsequently cleaved into α-MSH, β-MSH, and γ-MSH (53). The *fat/fat* mice carry a point mutation in *Cpe*, resulting in adult-onset obesity, altered thermoregulation and hyperglycemia (29, 54). In humans, mutations in *CPE* have also been identified in patients with severe obesity (55, 56). Initially, it was thought that their obese phenotype could be explained by defective processing of POMC into α-MSH. However, applying targeted genetic modification studies in mice suggest that defective POMC processing alone does not explain the phenotype observed in the *fat/fat* mice. POMC neuron-specific *Cpe* deficiency did not cause obesity in mice (57). Likewise, pancreatic β-cell-specific *Cpe* deficiency, affecting insulin processing, also did not result in spontaneous obesity (58). Thus, the mechanism by which Cpe contributes to energy homeostasis is more complex and likely involves defective processing of other peptides (53, 59). It should be noted that mice are deficient in β-MSH (60), thus β-MSH processing could be affected in humans carrying a *CPE* mutation.

Over the years significant numbers of whole-body knockout, tissue-specific knockout, and transgenic models have been developed that have provided more detailed insight into components of the leptin-melanocortin pathway regulating energy homeostasis. Likewise, human obesity-associated mutations have been introduced in mice and, when combined with in vitro studies, these can provide further insight into the signaling mechanisms involved (61). Furthermore, these models can aid in determining whether therapeutic interventions can rescue the obesity phenotype (62).

In summary, models of monogenic obesity in mice have the advantage of genetic precision and thus reproducibility. Since genetic modifications in mice can be derived directly from monogenic mutations found in humans, this makes them a powerful translational tool. However, this type of model may miss the additional impacts of the environment and the polygenic nature of human obesity. Additionally, although methods of producing animals with genetic modification are becoming more straightforward with the introduction of CRISPR-Cas9, it can still be expensive and time-consuming to generate genetically modified mice (Fig. 1).

## Diet-Induced Mouse Models of Obesity

Diet-induced obesity in mice is commonly used to model the obesogenic environment, better reflecting the interaction between environmental effects and the polygenic nature of human obesity. These diet-induced obese mouse models are therefore also used to study the response to anti-obesity therapies. A diet with a high fat content (HFD), ie, 45-60% fat compared to 4-6% fat in regular chow, is fed in most studies (63). However, diets rich in sugar, a combination of high fat and high sugar to model the effects of “Western” diets, and highly processed diets to model the effects of ultra-processed food consumption are also used (64-66). These diets have the advantage that their composition is standardized, in contrast to humans where diet is more variable (63, 67). The composition of the diet is important as in addition to homeostatic mechanisms controlling energy balance, hedonic mechanisms also are important. Hedonic feeding involves food intake driven by sensory perception or pleasure without metabolic demand and is mainly controlled by dopaminergic neurons in brain. However, the homeostatic and hedonic circuitry overlap and interact (reviewed in (68)). Indeed, it is suggested that the hedonic system can overrule the homeostatic system in both humans and mice, when a highly palatable diet is consumed (69, 70). An imbalance between homeostatic and hedonic mechanisms regulating food intake can contribute to the pathophysiology of obesity (69, 70). The balance between these 2 mechanisms is driven not only by the leptin-melanocortin system, as described above, but also by the gut hormone ghrelin (70). In addition to the hypothalamus, ghrelin and leptin receptors are expressed in reward centers in the brain, including the ventral tegmental area and the nucleus accumbens. Leptin suppresses whereas ghrelin enhances activity at these sites. There is evidence that they interact to modulate the mesolimbic dopamine pathway (71, 72). Ghrelin receptor (*Ghsr*) knockout mice have been shown to be resistant to HFD-induced obesity (73) and mice lacking active ghrelin (lacking the gene, *Goat*, that encodes ghrelin O-acyl transferase which acylates ghrelin) are resistant to obesity when fed a high-sugar diet (74). This suggests that, in addition to reduced homeostatic eating, their hedonic drive to eat palatable food is also suppressed in the absence of the ghrelin system. The ghrelin system, however, is complex: certain strains of *Ghsr* knockout mice do not display altered appetite/eating behaviors and knockout of ghrelin generally does not alter food intake among different strains (75).

Interestingly, the choice of inbred mouse strain will influence the response to a dietary challenge. The commonly used C57Bl6/J strain, but also the 129X1/Sv strain, are very susceptible to diet-induced obesity. In contrast, the SWR/J and BALB/cJ are examples of obesity-resistant strains (76). Further analysis showed that the diet-induced weight gain in obesity-prone strains was not driven by hyperphagia, but rather by a diminished amplitude of daily rhythm of eating behavior, which was unaffected in the obesity-resistant strains (77, 78). Thus, the genetic background not only influences the response of genetically modified mice to a dietary challenge but can also be exploited to identify the mechanisms underlying diet-induced obesity, including circadian eating behavior.

Overall, the effects of both genetic background and diet found in human obesity can be modeled with great similarity using mice in which obesity has been induced by diet. It is also a cost-effective and rapid method to induce obesity. However, the responses of mice to specific diets can be quite variable, even when using inbred strains (eg, weight gain and development of insulin resistance (66)), and there is often little conformity among different studies in types of diet used and period of time of exposure (Fig. 1).

## Confounding Factors

Our knowledge of the mechanisms that underly the development and consequences of obesity has been greatly advanced by the use of these mouse models. Despite this, it is important to acknowledge that they do have some limitations with respect to translatability to humans. However, major advantages compared to human studies lie in standardization of both their housing conditions and genetic background. This allows for careful dissection of the contribution of confounding factors to the susceptibility of obesity.

Here, we focus on 2 of these confounding factors, namely sexual dimorphism and differences in rates of energy expenditure.

### Metabolic Sexual Dimorphism

Historically, most of our knowledge is based on results obtained in male mice. However, upon inclusion of female mice in obesity research, it has become clear that there are striking differences between the sexes. For example, females are less susceptible to diet-induced obesity, showing weight gain at a slower pace and remaining more glucose tolerant than male mice (79, 80). Based on these and other studies it is suggested that females may have a better metabolic flexibility to adapt to changes in energy availability (81, 82). Interestingly, in humans, more women than men tend to be obese. Yet men have a higher risk of obesity-related metabolic diseases, despite the higher body fat percentage in women (83). The sex dimorphism in adipose tissue distribution, with women having more subcutaneous white adipose tissue and men more visceral white adipose tissue, is suggested to account for this difference in metabolic risk (84). Sex-dependent responses in food intake, locomotor activity, and energy expenditure are also observed (85), illustrating the added value of including indirect calorimetry assessment for deep metabolic phenotyping in mouse studies. Part of these sex differences in metabolism are attributed to differences in sex chromosomes and exposure to different levels of sex steroid hormones (reviewed in (86-88)).

There are examples of mouse models where specific genes influence this sex-dependent effect on metabolic function. Diet-induced obesity has been linked to hypothalamic neuronal damage and inflammation in both humans and rodents (89). This response, where high-fat diet causes rapid accumulation of pro-inflammatory, cytokine secreting, microglia in the hypothalamus, is limited to males, whereas females are resistant to microglial activation (90). This sex-dependent response is abolished upon manipulation of the protective chemokine C-X3-C motif ligand 1 (Cx3cl1) and its receptor (Cx3cr1) signaling in hypothalamic microglia. *Cx3cr1* deficiency in female mice resulted in a male-like, pro-inflammatory response, and central administration of Cx3cl1 in male mice induced a female-like response (91). This study suggests that CX3CR1 signaling is a sex-dependent molecular switch that regulates vulnerability to diet-induced obesity and that microglia are sexually dimorphic. A recently described example of a single gene acting sex-dependently to modulate body weight and the metabolic response to diet-induced obesity is C1q/TNF-related protein 10 (*Ctrp10*), which is nutritionally regulated in the brain and peripheral tissues (92). On a low-fat diet, loss of *Ctrp10* resulted in an obese phenotype with increasing age in female mice only. When fed an HFD, *Ctrp10* deficiency caused rapid weight gain in female mice, comparable to male mice (92). In both studies the sexual-dimorphic effects of the genes were found unlikely to be estrogen-driven, leaving the mechanism(s) still to be elucidated.

These examples highlight the importance of including both male and female mice in metabolic research. This is particularly relevant to understanding human metabolism in which sexual dimorphism is clearly evident, and which results in sex-dependent responses to anti-diabetic and anti-obesity drugs (82). Moreover, there is evidence of conservation of sex-dependency of the CX3CR1 signaling pathway (93) and of CTRP10 in relation to metabolism in humans, underscoring the translatability of these mouse studies to human physiology (91, 92).

### Energy Expenditure and Thermogenesis

A clear metabolic difference between humans and mice concerns the distribution of the components that comprise total energy expenditure (TEE). These include basal metabolic rate, diet-induced thermogenesis, and activity-induced energy expenditure. In sedentary humans, basal metabolic rate contributes 60% to 70% of TEE, while in single-housed mice this is only 30% to 35%. Spontaneous physical activity contributes between 20% and 25% to TEE in humans, whereas in mice this is at least 40%. Although the percentage of diet-induced thermogenesis does not differ between species (5%-10%), in mice a significant proportion of the TEE (20%-30%) is generated by cold-induced thermogenesis, also known as adaptive thermogenesis (94, 95). This percentage can be reduced by group housing or presence of nesting material, allowing mice to huddle, but yielding more variability in study results (96). Many studies in mice are performed at room temperature (20-23 °C), which is below their thermoneutral zone (∼30 °C) (97). In contrast to humans, maintaining the core body temperature in mice is predominantly regulated by BAT (96, 97). With cold exposure being a known activator of BAT by inducing uncoupling protein-1 (*Ucp1*) expression (98), the standard housing temperature of 20 to 23 °C can be considered a cold challenge. Indeed, compared to 30 °C, at 22 °C the TEE is ∼35% higher (99, 100). Moreover, mice housed at thermoneutrality become more obese than mice at 22 °C (101, 102), have decreased *Ucp1* expression, and an accelerated metabolic inflammatory profile (103, 104). Surprisingly, *Ucp1*-deficient mice had a normal body weight and were resistant to diet-induced obesity, when housed at room temperature (105). However, kept at thermoneutrality, these mice gained weight and became susceptible to HFD-induced weight gain (106). It is suggested that in addition to Ucp1-dependent thermogenesis, Ucp1-independent mechanisms also contribute to thermogenesis. These mechanisms, in which fibroblast growth factor 21 (FGF21) plays an important role, appear sufficient to compensate for *Ucp1* deficiency at room temperature. Only in the absence of both Ucp1 and Fgf21 do mice become obese upon HFD (107). At thermoneutrality, this Ucp1-independent mechanism is not activated (107) explaining, at least in part, the susceptibility to obesity in *Ucp1*-deficient mice.

It is suggested that the difference in ambient temperature between humans and mice could, in part, explain why anti-obesity drugs that induced weight loss in mice, failed to be successful in humans. However, when testing the efficacy of 5 anti-obesity drugs at 22 °C and 30 °C in diet-induced obese mice, Jacobsen et al (108) showed that the effect on weight loss appeared independent of the ambient temperature for some of these drugs (GLP-1 and hFGF21), while others showed opposite effects, being more potent at 22 °C (GDF15) or 30 °C (PYY). Thus, currently there is no clear-cut answer available as to which ambient temperature for mice yields best translatability to humans. Differences in Ucp1-dependent and Ucp1-independent mechanisms may contribute to this. An answer to this question is also complicated by the finding that preference for ambient temperature is sexually dimorphic (109). Female mice prefer a higher temperature than male mice, particularly in the inactive phase, which appears to be independent of sex steroid hormones in adult mice (110, 111). Interestingly, in contrast to male *Ucp1*-deficient mice, female *Ucp1*-deficient mice were not resistant to diet-induced obesity when housed under mild cold conditions (18 °C) (112). Thus, females may be more cold-sensitive, and this may also explain why female rodents have higher BAT mass and display differences in the BAT transcriptome at 22 °C (113, 114). Also in humans, women are suggested to have a 2-fold higher prevalence of active BAT (115, 116).

Sex-dependent effects of thermoneutrality on energy balance have been reported for several mouse models. Morris et al (117) showed that high-fat, high-sugar diet–induced weight gain was accentuated at thermoneutral temperature in male mice, whereas in female mice weight gain was unaffected by temperature. Both male and female kisspeptin receptor (*Kiss1r*) knockout mice develop obesity when housed at 22 °C, but at thermoneutral conditions (30 °C), the obese phenotype was attenuated in female mice only (118). Opposing effects were reported in mice with *CD47* deficiency in brown adipocytes, where age-related weight gain at both 22 °C and 30 °C was prevented only in male mice (119). These examples illustrate that there are many layers that influence energy homeostasis and to entangle these layers including thermoneutral conditions and sex in an experimental design may provide a deeper understanding of the mechanisms controlling energy homeostasis.

## Integrative Approaches to Translate Non-Coding Genome-Wide Association Study Signals of Obesity to Relevant Mouse Models

Monogenic mouse models of obesity have been very informative in providing insight into the regulatory mechanisms of energy homeostasis. However, the polygenic nature of human obesity brings new challenges to the identification of underlying biological pathways. Genome-wide association studies (GWASs) have identified over 900 loci associated with BMI, each with small effect sizes (120). Interestingly, genetic studies of both monogenic and polygenic obesity pinpoint to hypothalamic control of body weight regulation as many of the genes in these loci are expressed in the brain (121). Yet, determining their functional consequence is challenging since more than 80% of GWAS-identified loci are located in non-coding regions of the genome, suggesting that they influence gene expression rather than protein function (122). This also implies that their functional effect may be tissue specific (123). This complicates the use of animal models as many non-coding regions are species specific (124). However, recent advances in epigenetic profiling technologies to assess DNA methylation (MeDIP-seq), histone modifications (ChIP-seq), chromatin accessibility (ATAC-seq), higher order organization (HiC) combined with advanced bioinformatic tools may integrate GWAS results with epigenetic annotations. Such an integrative approach could be applied to identify those GWAS signals with a high level of conservation between human and mouse and use these to generate informative mouse models.

Studies comparing mouse and human multi-omics data suggest interspecies conservation that can be explored to unravel the involvement of identified loci in obesity-related pathways (125). Based on reanalysis of RNA sequencing data from multiple tissues from human and mice, collected by the mouse ENCODE consortium, it was suggested that gene expression data tend to cluster by tissue rather than by species (126). However, conflicting results have been published, as previous studies reported considerable RNA expression differences between mice and humans (127). Breschi et al (128) suggest that, based on expression data of multiple species, there is likely a continuum in the variation in gene expression, with on the one hand genes varying more across tissue type and less across species, and on the other hand genes varying more across species and less across tissue type. Indeed, results from the ENCODE consortium suggest that there is a high degree of functional conservation in the transcription factor networks between human and mice (129). However, specific locations of transcription binding sites may differ based on PPARγ and CTCF localization maps assessed in human and mouse adipocyte cell lines (130). The majority of the non-mapped transcription binding sites were located in rodent-specific transposable elements, suggesting that these elements likely evolved after the divergence of the species. However, when human and mouse binding sites could be mapped, it correlated with the presence of conserved motifs and open chromatin marks (130). This information could be leveraged to select those GWAS signals of BMI residing in shared conserved motifs for functional analysis in mouse models.

Interestingly, conserved transcription factor binding sites appear enriched for GWAS signals (131, 132). In line with this, Breschi et al (128) found that genes whose expressions displayed organ-dominated clustering tended to be associated with diseases and GWAS loci. Importantly, when analyzing human and mouse co-expression maps from GeneFriends, genes expressed in the brain showed a high degree of conservation in terms of co-expression network connectivity (133). Furthermore, in terms of conservation of co-expression, the most conserved gene sets were related to metabolic disorders (133).

This might suggest that analysis of interspecies conservation is particularly relevant to disentangle the polygenic nature of metabolic control. Comparisons of tissue-specific methylation patterns between rat, mouse and human suggest that a minimum of 11% to 37% of these patterns are conserved in the 3 tissues (blood, brain, and sperm) analyzed, and this increased to 57% when orthologous sequences were compared (134). This study did not include metabolic-related tissues, and thus the level of epigenetic conservation may be different in hypothalamus and adipose tissue, for example. While this percentage of tissue-specific methylation patterns may seem low, these patterns were enriched for conserved transcription factor binding elements (134). Therefore, epigenetic conservation could be utilized to prioritize GWAS signals with the highest potential of effective translatability to mouse models.

Leveraging interspecies epigenetic profiles can also be of interest to identify biological pathways affected by environmental triggers. In an interesting study applying this approach, diet-induced epigenetic changes in mice were integrated with epigenetic changes in human adipose tissue samples from the same subjects before and after Roux-en-Y gastric bypass to identify epigenetic conserved regions (135). These cross-species obesity-associated regions were shown to overlap with several type 2 diabetes risk loci. Subsequent functional analyses by overexpression or knockdown of genes linked to these loci in a mouse adipocyte cell line implicated a potential role for several of the genes in insulin sensitivity regulation (135). Combining such data with single-nucleus RNA sequencing and spatial transcriptomic data—for example, by comparing the mouse Hypomap and human HYPOMAP—can provide another perspective to identify interspecies neuronal conservation or heterogeneity (136). Therefore, when generating animal models to study regulatory variants associated with energy homeostasis, it may be important to take epigenomic conservation into account. However, for these experiments to be successful, that is, to prioritize relevant targets, multi-omics data of relevant human and mouse tissues are needed.

## Conclusions

Over the years, a variety of mouse models of obesity have each uniquely enhanced our insight into the cellular and molecular mechanisms regulating energy homeostasis. These mouse models have allowed us to study the interaction between genetic and environmental factors under controlled conditions. However, although conserved mechanisms exist in humans and mice, there are also differences that should be taken into account when translating results. Advanced genomic technologies and bioinformatics can aid in addressing these species differences as they provide new tools to translate GWAS findings into meaningful biological insights. Although induced pluripotent stem cell technology and organ-on-a-chip technologies are promising developments to reduce the number of animals in research, they are currently not sufficiently advanced to model the complex, multi-level interactions regulating energy homeostasis in whole animals, particularly at the level of the brain. To improve our ability to translate the outcomes of mouse studies to humans, carefully designed experiments that account for confounding factors, such as sex, age, environmental temperature, and type and duration of diet, are required.

## Disclosure

J.A.V. has received royalties from AMH assays, paid to the institute/lab with no personal financial gain. P.J.D.D. declares no conflict of interest.

## Data Availability

Data sharing is not applicable to this article as no datasets were generated or analyzed for this review.

## Abbreviations

α-MSH: α-melanocyte stimulating hormone;
AgRP: agouti-related peptide
ASP: agouti signaling protein
BAT: brown adipose tissue
BMI: body mass index
CPE: carboxypeptidase E
GPCR: G protein–coupled receptor
GWAS: genome-wide association study
HFD: high-fat diet
MC4R: melanocortin-4 receptor
POMC: pro-opiomelanocortin
TEE: total energy expenditure
